# Scientific Production in Dentistry: The National Panorama through a Bibliometric Study of Italian Academies

**DOI:** 10.1155/2020/3468303

**Published:** 2020-08-05

**Authors:** Felice Lorusso, Francesco Inchingolo, Antonio Scarano

**Affiliations:** ^1^Department of Medical, Oral and Biotechnological Sciences, University of Chieti-Pescara, Via dei Vestini, 31, 66100 Chieti, Italy; ^2^Department of Interdisciplinary Medicine, University of Bari “Aldo Moro”, 70121 Bari, Italy

## Abstract

**Background:**

The academic scientific research in the field of dentistry has rapidly increased in the last 20 years under the pressure of the multidisciplinary technological advancements and the growing demand for new predictable and cost-effective techniques and materials. The aim of the present investigation was to analyze the academic scientific production conducted by Italian Academies and Dental Schools.

**Methods:**

The list of MED/28 academic researchers, associate and full professors, and academic affiliations was collected from the national database of CINECA to evaluate the scientific output of the Italian Universities. The complete list of scientific contributions and the bibliometric parameters were recorded in the Scopus database.

**Results:**

The scientific production of 37 Italian Universities, 416 researchers, and 23689 papers was evaluated. The measurement of total academic papers, citations, h-index, and relative citation ratio (RCR) was calculated. The study data showed an increase of the academic scientific production over the last 5 years.

**Conclusions:**

The results presented show how scientific research is increasingly pursued by dental clinicians.

## 1. Introduction

In recent years, the progress of scientific research in medicine and dentistry is growing due to the technological advances in techniques and materials that are improving the quality of life [1–3].

The academic scientific research has gradually increased in the last years following the world trend and today represents an important element for university academic careers in the bibliometric disciplines [4, 5]. Scientometrics is the discipline that evaluates the quality of the scientific production by techniques and indicators able to measure the bibliographic data and the process of scholarly communication [6–9].

Moreover, the bibliometric research provides a key role for the evaluation of the scholarly chain by measuring methodologies of the scientific productivity of researchers, academies, and scientific associations [10–12]. An extended national bibliometric evaluation represents a valuable methodology able to create a demographic and trend analysis [13–15].

In fact, the evaluation of the scientific production of a single researcher or an institution can be done through access to one of the dedicated databases existing in the network [16, 17]. One of the main problems of those approaches is represented by the potential systematic bias [11, 16, 18, 19].

Several assessment parameters have been proposed for this scope, such as the journal impact factor citation count, the h-index, and the contemporary h-index that are based on paper citation rate calculation [8, 10].

Dentistry discipline is focused on the prevention, diagnosis, and treatment of oral diseases and disorders and maintenance of oral health [3, 20–22]. This clinical activity is centered on hard and soft tissues, oral mucosa, teeth, maxillofacial bones, temporomandibular, and other supporting structures [23–26].

Moreover, the therapeutic approaches, materials, and protocols need to be convalidated, updated, and constantly revised to increase the predictability of the outcomes in clinical practices [27, 28].

The aim of the present investigation was to perform a bibliometric analysis of the scientific academic production of the public and private Italian Universities.

## 2. Materials and Methods

### 2.1. Selection of the Sample

The bibliometric quantitative evaluation and content analysis was performed in accordance with the Standards for Reporting Qualitative Research (SRQR) [27].

A list of academic researchers of the Italian Universities was obtained from the national institutional database CINECA (https://www.cineca.it) and recorded by two expert specialists (F.L.) into a special dedicated electronic database by the Excel software package (Microsoft Corporation, Redmond, Washington, USA). The recordings were classified and indicized as researchers, associate professors, and full professors affiliated to the academic medical-disciplinary sector odontostomatological diseases (MED/28) for demographic evaluations. For the present investigation, also the position of a researcher at a determined time was considered for the bibliometric evaluation.

### 2.2. Data Collection

The study data were found and recorded from March 2 to April 8, 2020, from the researcher list of Italian Academics, then analyzed and included in this study.

The database chosen for the bibliometric data evaluation was SciVerse® Scopus (https://www.scopus.com). The bibliometric data collection was performed by two operators with experience in the field of literature search (L.F. and A.S.). The author search was performed on the electronic database and included the following data: surname and initial of first name. The authors entered only the initial of the name to avoid possible loss of data, due to the fact that in some publications the full name of the author does not appear. In case of a disambiguation mismatch, the results of the research were excluded. For the bibliometrical analysis, all contribution types recorded in the database (such as proceedings, review, article, and letter) were considered.

### 2.3. Scientific Production Assessment

For each academic author search, the total number of papers, total citations, and h-index was computed. Moreover, the last ten-year publications were considered to evaluate the trend in scientific production. All data were included in a spreadsheet Office Excel 2007 (Microsoft Corporation) and processed to calculate the mean, the median, and the interquartile range (Irq) and the percentage change between the individual values where required. The most cited papers for each academic professional were collected for the academic cumulative mean, and the indexed papers, h-index, and total citations were calculated.

## 3. Results

### 3.1. Study Population

For the present investigation, a total of 37 Italian universities, 416 academics (153 researchers, 175 associate professors, and 88 full professors), and 23538 indexed papers were evaluated for demographic and statistical analysis (Table 1).

The distribution of the academics is presented in Table 2 (total range between 29 and 2).

The researchers ranged from 29 to 0 (mean: 5.5 ± 4.7), the associated professors ranged between 18 and 3 (mean: 7.1 ± 4.1), and the full professors between 29 and 2 (mean: 4.2 ± 2.7).

### 3.2. Academics Scientific Production

**Figure 1:**
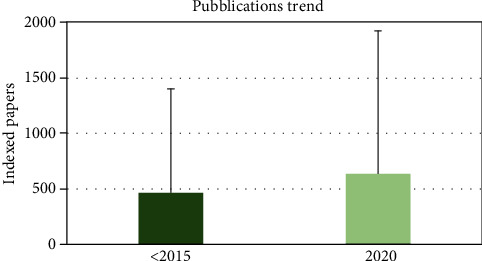
Scientific production trends of the academics in the last 5 years.

An increase of the scientific production was reported during the last 5 years for all academics (2015-2020) (Figure 1).

A full professor mean h-index was reported higher if compared to associate professors and researchers, while the researchers' increase of indexed papers was 53.4%, the associate professors' increase was 57.8%, and the full professors' increase was 45.5%.

**Figure 2:**
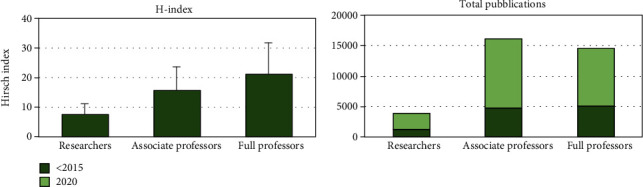
Total paper production and mean h-index of the academics evaluated.

**Figure 3:**
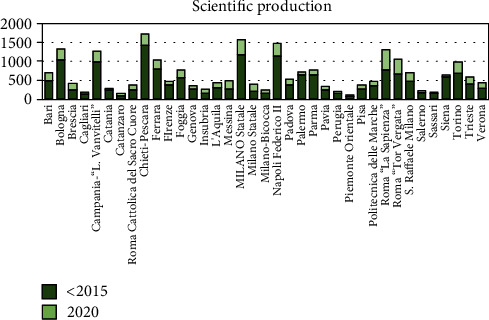
Scientific production trends referring to the 37 universities evaluated.

An increase of the academic scientific papers was reported in the last 5 years, with an augmented production index ranging between 52.4% and 91.7% (Figure 2) and a distribution of the publications between the three professional categories (Figure 3).

The summary of the bibliometric parameters of the Italian schools of dentistry are presented in Table 3, with the total count of indexed papers, h-index, total citations, and cumulative most cited paper value.

**Figure 4:**
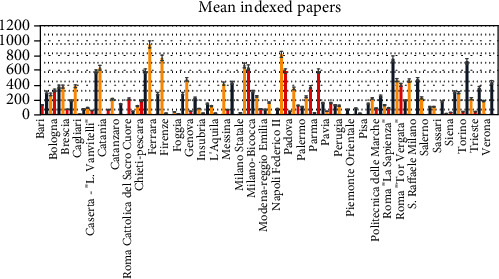
Indexed papers distribution of the researchers (red), associates (blue), and full professors (yellow) investigated.

**Figure 5:**
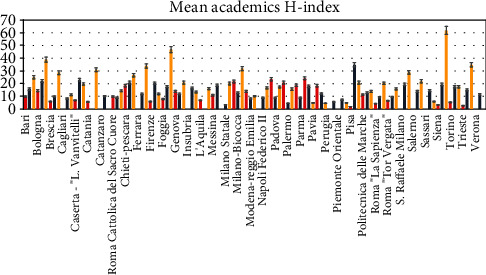
Mean h-index distribution between the researchers (red), associates (blue), and full professors (yellow).

**Figure 6:**
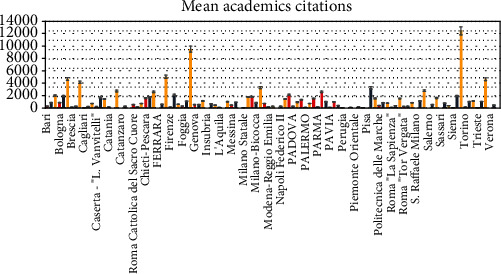
Mean citation amount distribution of the academic professionals referring to the universities investigated. Researcher (red), associated (blue), and full professors (yellow).

A heterogenicity of the amount of indexed paper (Figure 4), mean h-index (Figure 5), and mean citations count (Figure 6) are reported between the academic categories of the universities evaluated.

## 4. Discussion

The scholastic institution in the field of dentistry in Italy presents a more recent historical course if compared to the other medical sectors [28].

In Italy, the dentistry profession is currently practiced by three different figures: the graduate in Medicine and specialized in Odontostomatology; the graduate in Medicine and Surgery who is not a specialist but registered in the National Register of Dentists; and the graduate in Dentistry. In the same way, the researchers' careers afferent to the academic medical-disciplinary sector, odontostomatological diseases (MED/28), require a degree in medicine and dentistry.

Nowadays, the clinical and research activity in dental practice covers several different specialties such as oral surgery and implantology, odontostomatology, orthodontics, pediatric, restorative, and prosthetic dentistry. As a result, dental research has shown a worldwide increase of scientific production output in the last decades [29].

Pulgar et al. reported a quantitative analysis of the scientific production on electronic database, investigating Dentistry, Oral Surgery, and Medicine (DOSM) publications and Non-DOSM production. The percentage of dental papers, including surgery manuscripts, compared to total production was 0.89% during the last three decades, with a Non-DOSM/DOSM ratio of 17% [29].

Moreover, the Italian scientific production was considered among the top 20 countries with an increase of 4.43% of DOSM publications during the same period [29].

In the present investigation, the academics of the Italian Universities registered in the national institutional database were considered for evaluating the scientific production trend.

However, this methodology does not consider the scientific contribution offered by the private practitioners and hospital dental employees, who represent a consistent part of the dental health care in Italy [30].

The present investigation was not extended to health workers of hospitals and public assistance structures, where the bibliometric parameters are not institutional indicators for the careers of the clinicians in the public healthcare structures.

In this way, the adoption of new research strategies of quality scientific production could improve the researchers' activity in studying new approaches and therapeutic treatments for oral and jaw diseases and for a better knowledge of their etiopathogenesis [1, 31, 32].

In a previous research, Zizzari et al. investigated the scientific production of 252 active members of Italian associations of Oral Surgery throughout three periods of 5 years each, covering a total of 15 years [33]. The study showed that the nonacademic scientific production produced from 2886 to 5679 papers during the period between 2003 and 2008, 7865 from 2009 to 2013, with an increase of 172.52% manuscripts.

One of the most important limits of the research design is represented by the systematic research bias [29, 34]. In fact, the disambiguation of authors represents the weak point of the of the present methodology.

Moreover, a comparison of the investigation results with the international academic scientific production is a possible perspective, but the high risk of bias is present in relation to the extensive differences between the nations' academic systems and institutional affiliations in medicine and dentistry. Probably the presence of a common European and international researchers register can facilitate the check of the academics for a supranational bibliometric comparison.

Scopus provides the most complete database with the largest scientific bibliography and citations system, with over 18000 journal sources registered, covering several fields, such as medicine, engineering, humanities, and social disciplines [19, 35].

In the present study, the institutions with an increased amount of academics showed the higher level of scientific production, in terms of total published papers. On the contrary, the other quality production indexes such as citation count and h-index showed a great heterogenicity of the output, with a production index that exceeded 90% in the last 5 years.

However, clinical research in dental practice of the Italian academics concerned the different disciplines of dentistry: oral surgery and implantology, odontostomatology, orthodontics, pediatric, restorative, and prosthetic dentistry. In fact, the recent research activity in dentistry showed a significant increase of scientific production output in the last decades, following the advances in new materials, clinical protocols, technical procedures, and technologies in the relative disciplines.

Today, the scientific production represents an important element of evaluation for the university researchers' careers in the bibliometric disciplines and probably a substantial incentive to enhance the present activity.

Moreover, the bibliometric parameters used do not represent the outline of the years of activity of the individual academics that could influence the quality trends of the younger researchers [36–38].

In this way, a normalized citation index should be introduced to overcome this activity difference and reduce the potential confounding factor between the researchers, associate professors, and full professors to a more equal evaluation trend [6, 12, 15, 30].

## 5. Conclusions

The existing databases represent valuable tools for measuring the quality and quantity of the institutional scientific production according to an appropriate interpretation of the data, with a growth in the last 5 years in the trend of academic activity with a high scientific-impact indices output.

## Figures and Tables

**Table 1:** Demographic evaluation of the academics of the 37 Italian Universities evaluated.

Academic positions	Total	Total papers	Mean h-index	Total citations	Papers published (2015-2020)
Researchers	153	2666	7.4 ± 5.1	29441	1425
Associate professors	175	11372	15.7 ± 8.4	175378	6583
Full professors	88	9500	21.2 ± 11.4	168089	4369

**Table 2:** Distribution of the academics in the 37 Italian Universities evaluated.

Universities	Researchers	Associate professors	Full professors
Bari	3	4	2
Bologna	9	4	3
Brescia	5	6	2
Cagliari	1	3	2
Campania-“L. Vanvitelli”	4	7	5
Catania	6	0	1
Catanzaro	1	3	0
Roma Cattolica del Sacro Cuore	5	3	2
Chieti-Pescara	5	6	8
Ferrara	0	5	3
Firenze	4	4	1
Foggia	2	4	1
Genova	2	4	1
Insubria	2	2	2
L'Aquila	2	0	5
Messina	3	6	0
Milano	6	12	6
Milano-Bicocca	4	0	1
Modena e Reggio Emilia	3	4	0
Napoli Federico II	8	12	7
Padova	2	4	1
Palermo	8	4	4
Parma	5	3	2
Pavia	5	2	3
Perugia	4	0	3
Piemonte Orientale	0	2	1
Pisa	2	1	3
Politecnica delle Marche	2	5	2
Roma “La Sapienza”	12	18	4
Roma “Tor Vergata”	20	5	5
S. Raffaele Milano	1	7	1
Salerno	0	1	1
Sassari	2	3	1
Siena	2	5	1
Torino	6	12	3
Trieste	6	6	1
Verona	1	8	0

**Table 3:** Summary of the Italian Academies investigated (tot: total cumulative count; mean: average amount; sd: standard deviation, Icq: interquartile range).

Universities		Indexed papers	h-index	Citations	Papers (2015-2020)	Most cited paper
Bari	Tot	707.0	153.0	8780.0	218.0	724.0
	Mean	70.7	15.3	878.0	21.8	72.4
	sd	51.1	6.7	829.1	14.1	48.3
	Irq	63.0	9.5	979.3	19.5	78.5

Bologna	Tot	1334.0	321.0	33299.0	292.0	2661.0
	Mean	95.3	22.9	2378.5	20.9	190.1
	sd	69.6	14.0	2848.5	17.8	160.5
	Irq	77.8	16.3	2019.8	26.0	111.8

Brescia	Tot	408.0	125.0	4606.0	156.0	841.0
	Mean	31.4	9.6	354.3	12.0	64.7
	sd	21.5	4.6	407.9	9.9	61.9
	Irq	28.0	6.0	278.0	13.0	22.0

Cagliari	Tot	182.0	59.0	2162.0	60.0	350.0
	Mean	30.3	9.8	360.3	10.0	58.3
	sd	17.4	4.6	302.9	8.5	34.3
	Irq	20.8	6.3	348.5	13.3	44.5

Campania- “L. Vanvitelli”	Tot	1272.0	282.0	20096.0	284.0	2274.0
	Mean	84.8	18.8	1339.7	18.9	151.6
	sd	55.0	8.1	1006.0	17.8	108.1
	Irq	65.0	11.5	1406.0	14.5	194.0

Catania	Tot	283.0	54.0	3390.0	32.0	276.0
	Mean	56.6	10.8	678.0	6.4	55.2
	sd	87.8	11.6	1168.2	13.2	37.9
	Irq	18.0	6.0	346.0	1.0	60.0

Catanzaro	Tot	143.0	31.0	873.0	50.0	114.0
	Mean	47.7	10.3	291.0	16.7	38.0
	sd	8.5	2.3	64.6	8.4	5.6
	Irq	8.5	2.0	61.5	7.5	5.5

Roma Cattolica del Sacro Cuore	Tot	381.0	98.0	5040.0	140.0	1965.0
	Mean	42.3	10.9	560.0	15.6	218.3
	sd	18.4	4.5	410.2	10.6	285.8
	Irq	24.0	5.0	706.0	15.0	252.0

Chieti-Pescara	Tot	1729.0	391.0	33835.0	294.0	2995.0
	Mean	96.1	21.7	1879.7	16.3	166.4
	sd	77.6	10.4	2088.3	17.5	119.7
	Irq	68.5	14.8	1590.3	18.3	190.5

Ferrara	Tot	1054.0	162.0	18314.0	247.0	1036.0
	Mean	131.8	20.3	2289.3	30.9	129.5
	sd	146.9	14.5	3210.8	27.2	83.1
	Irq	99.3	11.0	1318.0	43.5	82.5

Firenze	Tot	466.0	100.0	9317.0	90.0	923.0
	Mean	77.7	16.7	1552.8	15.0	153.8
	sd	92.1	11.9	2528.0	25.1	167.7
	Irq	18.5	3.5	348.0	4.8	73.5

Foggia	Tot	778.0	126.0	14294.0	202.0	758.0
	Mean	129.7	21.0	2382.3	33.7	126.3
	sd	173.0	14.3	3549.0	38.2	77.4
	Irq	23.5	11.8	1217.3	24.3	77.0

Genova	Tot	360.0	83.0	3857.0	101.0	320.0
	Mean	60.0	13.8	642.8	16.8	53.3
	sd	28.9	6.9	403.7	9.5	28.3
	Irq	39.0	3.8	410.8	8.3	11.0

Insubria	Tot	266.0	60.0	2366.0	122.0	186.0
	Mean	66.5	15.0	591.5	30.5	46.5
	sd	21.5	2.9	296.5	14.4	25.5
	Irq	25.0	2.5	358.5	11.0	24.5

L'Aquila	Tot	443.0	88.0	5189.0	137.0	368.0
	Mean	73.8	14.7	864.8	22.8	61.3
	sd	43.7	7.4	675.4	17.8	21.3
	Irq	61.8	13.8	1169.8	24.3	32.8

Messina	Tot	500.0	135.0	6639.0	235.0	422.0
	Mean	62.5	16.9	829.9	29.4	52.8
	sd	52.6	8.7	737.2	31.5	17.8
	Irq	26.5	7.5	606.3	12.0	16.3

Milano	Tot	1581.0	352.0	30365.0	393.0	2675.0
	Mean	87.8	19.6	1686.9	21.8	148.6
	sd	67.5	12.3	1615.1	21.5	119.7
	Irq	61.3	16.0	2160.0	25.8	170.0

Milano-Bicocca	Tot	391.0	66.0	4080.0	186.0	466.0
	Mean	78.2	13.2	816.0	37.2	93.2
	sd	77.7	9.9	1064.7	54.7	56.9
	Irq	45.0	7.0	584.0	9.0	68.0

Modena e Reggio Emilia	Tot	237.0	58.0	1584.0	88.0	248.0
	Mean	39.5	9.7	264.0	14.7	41.3
	sd	8.6	2.9	149.0	9.7	11.4
	Irq	11.0	3.0	117.0	12.8	12.5

Napoli Federico II	Tot	1487.0	330.0	27739.0	348.0	6555.0
	Mean	78.3	17.4	1459.9	18.3	345.0
	sd	53.1	8.5	1710.8	14.9	917.9
	Irq	68.0	13.5	1625.0	20.0	157.0

Padova	Tot	527.0	100.0	5518.0	143.0	455.0
	Mean	87.8	16.7	919.7	23.8	75.8
	sd	26.1	4.5	436.8	11.6	29.3
	Irq	14.8	5.0	478.0	17.8	27.5

Palermo	Tot	730.0	166.0	10123.0	81.0	1154.0
	Mean	52.1	11.9	723.1	5.8	82.4
	sd	52.3	9.8	1220.1	7.5	56.8
	Irq	50.5	10.0	508.0	5.8	71.8

Parma	Tot	777.0	118.0	10109.0	126.0	913.0
	Mean	129.5	19.7	1684.8	21.0	152.2
	sd	127.7	7.1	1542.6	11.8	94.6
	Irq	80.3	8.0	922.3	7.0	144.8

Pavia	Tot	341.0	89.0	3581.0	96.0	516.0
	Mean	42.6	11.1	447.6	12.0	64.5
	sd	29.2	5.9	372.6	11.4	31.7
	Irq	49.3	7.3	338.5	17.5	34.5

Perugia	Tot	198.0	31.0	687.0	50.0	189.0
	Mean	33.0	5.2	114.5	8.3	31.5
	sd	29.5	3.5	110.1	7.6	29.7
	Irq	27.8	5.8	185.3	11.5	40.8

Piemonte Orientale	Tot	108.0	20.0	482.0	39.0	85.0
	Mean	36.0	6.7	160.7	13.0	28.3
	sd	18.0	1.5	136.6	8.9	23.3
	Irq	17.5	1.5	131.0	8.5	21.5

Pisa	Tot	373.0	100.0	8028.0	103.0	802.0
	Mean	74.6	20.0	1605.6	20.6	160.4
	sd	52.1	12.3	1265.2	15.8	113.5
	Irq	8.0	10.0	1511.0	19.0	183.0

Politecnica delle Marche	Tot	475.0	115.0	6705.0	114.0	887.0
	Mean	52.8	12.8	745.0	12.7	98.6
	sd	35.9	7.6	821.9	9.9	94.4
	Irq	49.0	8.0	742.0	18.0	66.0

Roma “La Sapienza”	Tot	1315.0	274.0	12994.0	539.0	1603.0
	Mean	48.7	10.1	481.3	20.0	59.4
	sd	47.0	6.8	657.5	20.8	48.8
	Irq	23.0	7.5	504.0	16.5	42.5

Roma “Tor Vergata”	Tot	1061.0	251.0	10298.0	391.0	2393.0
	Mean	36.6	8.7	355.1	13.5	82.5
	sd	39.7	5.6	399.8	15.3	82.5
	Irq	36.0	7.0	317.0	18.0	67.0

S. Raffaele Milano	Tot	709.0	165.0	10981.0	240.0	911.0
	Mean	88.6	20.6	1372.6	30.0	113.9
	sd	63.4	7.1	839.5	22.5	57.6
	Irq	37.8	12.0	960.0	27.5	79.0

Salerno	Tot	218.0	36.0	2206.0	45.0	237.0
	Mean	109.0	18.0	1103.0	22.5	118.5
	sd	1.4	5.7	769.3	12.0	108.2
	Irq	1.0	4.0	544.0	8.5	76.5

Sassari	Tot	194.0	50.0	2858.0	49.0	430.0
	Mean	38.8	10.0	571.6	9.8	86.0
	sd	49.0	8.9	748.8	13.2	75.8
	Irq	14.0	6.0	263.0	11.0	133.0

Siena	Tot	644.0	166.0	22226.0	53.0	1299.0
	Mean	80.5	20.8	2778.3	6.6	162.4
	sd	98.2	22.0	4437.1	8.4	142.5
	Irq	73.3	26.0	3163.5	7.3	199.8

Torino	Tot	984.0	281.0	16305.0	298.0	1937.0
	Mean	54.7	15.6	905.8	16.6	107.6
	sd	28.1	6.6	620.6	12.4	58.8
	Irq	32.8	10.0	833.0	22.5	79.3

Trieste	Tot	594.0	138.0	11181.0	188.0	2015.0
	Mean	54.0	12.5	1016.5	17.1	183.2
	sd	55.4	11.9	1523.0	16.0	260.2
	Irq	55.5	12.5	1046.0	18.0	190.5

Verona	Tot	439.0	93.0	4018.0	161.0	771.0
	Mean	54.9	11.6	502.3	20.1	96.4
	sd	41.0	3.6	143.6	19.8	65.0
	Irq	55.0	4.5	168.3	26.8	54.5
